# Palmitoylethanolamide, a Natural Retinoprotectant: Its Putative Relevance for the Treatment of Glaucoma and Diabetic Retinopathy

**DOI:** 10.1155/2015/430596

**Published:** 2015-11-18

**Authors:** Jan M. Keppel Hesselink, Ciro Costagliola, Josiane Fakhry, David J. Kopsky

**Affiliations:** ^1^University of Witten/Herdecke, Alfred-Herrhausen-Straße 50, 58448 Witten, Germany; ^2^Institute of Neuropathic Pain, Vespuccistraat 64-III, 1056 SN Amsterdam, Netherlands; ^3^Università degli Studi del Molise, Via Francesco De Sanctis, No. 1, 86100 Campobasso, Italy; ^4^Faculty of Medicine, Department of Pharmacology and Toxicology, American University of Beirut, P.O. Box 110236, Beirut, Lebanon

## Abstract

Retinopathy is a threat to the eyesight, and glaucoma and diabetes are the main causes for the damage of retinal cells. Recent insights pointed out a common pathogenetic pathway for both disorders, based on chronic inflammation. Palmitoylethanolamide (PEA) is an endogenous cell protective lipid. Since its discovery in 1957 as a biologically active component in foods and in many living organisms, around 500 scientific papers have been published on PEA's anti-inflammatory and neuron-protective properties. PEA has been evaluated for glaucoma, diabetic retinopathy, and uveitis, pathological states based on chronic inflammation, respiratory disorders, and various pain syndromes in a number of clinical trials since the 70s of 20th century. PEA is available as a food supplement (PeaPure) and as diet food for medical purposes in Italy (Normast, PeaVera, and Visimast). These products are notified in Italy for the nutritional support in glaucoma and neuroinflammation. PEA has been tested in at least 9 double blind placebo controlled studies, among which two studies were in glaucoma, and found to be safe and effective up to 1.8 g/day, with excellent tolerability. PEA therefore holds a promise in the treatment of a number of retinopathies. We discuss PEA as a putative anti-inflammatory and retinoprotectant compound in the treatment of retinopathies, especially related to glaucoma and diabetes.

## 1. Introduction

Different types of chronic eye pathologies share a common chronic inflammatory response, which induce in the affected tissues an immunopathological environment responsible for disease progression and a further tissue destruction and abnormal organ homeostasis [1–3]. Among these, it has recently been recognized that age-associated degenerative eye diseases such as glaucoma, age related macular degeneration and diabetic retinopathy, have strong immunological bases; in fact, also for these disorders (neuro)inflammation seems to be a common ground [1, 2, 4]. These new insights prompted us to explore and review the putative role of palmitoylethanolamide (PEA), a peroxisome proliferator-activated receptor alpha (PPAR-*α*) ligand that exerts anti-inflammatory, analgesic, and neuroprotective actions [5], for the treatment of (neuro)inflammation, especially related to glaucoma and diabetic retinopathy.

The retina as nervous tissue is highly vulnerable, especially the retinal ganglion cells (RGC), and quickly suffers damage due to a variety of disorders. Protection of the retinal cells against insults and the restoration of damaged tissue are therefore high on the list of ophthalmologists. Neuroprotective and restorative compounds are widely searched for by academia and the pharmaceutical industry. Compounds protecting cells against ischemic and reperfusion damage and immunological, toxic, and metabolic insults might have a therapeutic value for the treatment of retinopathies. There is a high medical need to identify such retinoprotective agents, especially for the treatment of retinopathies due to diabetes and glaucoma. Recently it has been recognized that one of the main pathogenetic mechanisms leading to diabetic and glaucomatous retinopathy has its fundament in chronic inflammation, leading amongst others to increased levels of inflammatory cytokines and activation of the NF-kappa-B pathway. This was the reason to review the relevance of palmitoylethanolamide for the prevention and treatment of such disorders, as PEA can both inhibit such chronic inflammatory cascades as well as protect neural cells against damage.

Starting in the early 80s of the 20th century, clinical trials evaluating putative cytoprotective new chemical entities, for instance calcium antagonists, lazaroids, and NMDA antagonists, did not succeed in identifying a therapeutically safe and useful compound [6]. Most compounds, which were cytoprotective in animal models, failed to be effective in humans [7]. In addition, a monotarget approach was proven not to be very useful as most morbid states are defined by a number of pathogenetic pathways. This is the reason why PEA as an endogenous agonist for PPARalpha, which engages multiple transduction systems in the cell to achieve its homeostatic effects, might be so useful. Palmitoylethanolamide (PEA) might be a retinoprotectant, as it has cytoprotective and immune (and glia-) modulating properties and is characterized by multiple tissue-targets (mast cells, glia cells, and retina cells) [8]. PEA lowers intraocular pressure, both in normal and in high-pressure glaucoma, as well as after laser iridotomy [9–12]. In an uveitis model, PEA inhibits inflammation and protects eye tissue [13]. There are also indications for a natural neuroprotective function of PEA in the retinal tissue [14, 15]. PEA's metabolism has been extensively explored [5, 16]. It is synthesized from precursors residing in the cell membrane via specific enzymes such as N-acyltransferase and N-acylphosphatidylethanolamine-preferring phospholipase D (NAPE-PLD). The termination of its action is dependent partly on fatty acid amide hydrolase (FAAH) and mostly on N-acylethanolamine-hydrolysing acid amidase (NAAA).

We will review and discuss in this paper the clinical and pharmacological data supporting PEA's use in glaucoma, especially related to the neuroinflammatory factors involved, followed by a discussion of PEA's anti-inflammatory, cytoprotective, and retinaprotective properties. Thereafter, we will present the data related to the inflammatory pathogenesis in diabetic retinopathy and the putative role of PEA in the treatment of such complications.

## 2. Palmitoylethanolamide in Glaucoma

Glaucoma is the second most important cause of blindness in the world, which lead to (i) retinal nerve fiber layer alterations; (ii) optic nerve head cupping; and (iii) typical visual field defect [17]. Elevated intraocular pressure (IOP) has been recognized as the major risk factor for the development of glaucoma and the only modifiable factor associated with the disease [18]. Strong evidence suggests that an early insult occurs to RGC axons at the optic nerve head. The exact mechanisms by which RGC axons are insulted and ultimately degenerate are not clear, although early neuroinflammatory responses seem to suggest a role of inflammation in glaucoma [19].

Lipid messengers and endocannabinoids from the PEA family are synthesized in ocular tissue and have been identified as intraocular pressure reducing compounds. PEA itself however cannot be classified anymore as a pure endocannabinoid; we prefer to define it as a lipid messenger or a lipid autocoid. In animal model anandamide, an example of a pure endocannabinoid (50 *μ*g to 1 mg) induced a significant decrease in intraocular pressure (IOP) within 1 hour after topical administration [20, 21]. The putative role of such protective molecules in the treatment of glaucoma was discussed already a decade ago [22]. With regard to PEA, its concentration is lower in the ciliary body of the eyes of glaucoma patients compared to normal [22]. In states of chronic inflammation, such as diabetic retinopathy and age-related macular degeneration, however, PEA levels were raised, which is explained as an effort of the body to reach homeostasis and downregulate inflammatory pathways [14]. In a model for uveitis exogenous PEA could significantly reduce inflammatory parameters [13]. PEA administration as a depot injection could also significantly increase PEA levels in the retina [23].

In a prospective, randomized, double-blind, crossover clinical trial, 42 patients with raised IOP, treated insufficiently with timolol 0.5% (IOP remained slightly increased, between 19 and 24 mmHg), received oral PEA or placebo for 2 months [11]. After a 2-month washout period patients entered the other treatment arm, during 1 month. PEA (600 mg/day) reduced IOP by 3.2 ± 1.3 mmHg at 1 month and by 3.5 ± 1.2 mmHg at 2 months. In the placebo group IOP was reduced by 0.4 ± 1.2 mmHg at 1 month and by 0.3 ± 1.3 mmHg at 2 months. The difference in IOP reduction was significant for both time points (*p* < 0.001).

In another randomized double-blind placebo-controlled, crossover study, 40 naive ocular hypertensive patients underwent endothelium-dependent flow-mediated dilation measurements (FMD) in addition to the intraocular pressure measurements. Patients were treated by either PEA (600 mg/day) or a matching placebo for three months. The first treatment period was followed by a two-month washout period. Subsequently patients crossed over to PEA or placebo for another three months. The conclusion was that treatment by PEA during 3 months reduced IOP and led to significantly improved FMD values in ocular hypertensive patients compared to placebo, by ameliorating peripheral endothelial function, and its positive effect lasted longer than the period of PEA consumption, as measured after 2 months of wash-out. No adverse events were recorded [10].

In another clinical study, intraocular pressure and visual field (VF) damage progression in normal-tension glaucoma (NTG) were evaluated in 32 patients and compared with a control group. Patients were randomized in a 1 : 1 ratio to receive PEA (600 mg/day) treatment for 6 months or no treatment for the same period. Best-corrected visual acuity, IOP, and visual field test were evaluated at baseline and at the end of the six-month follow-up. At six months, PEA treatment resulted in a significant IOP reduction (from 14.4 ± 3.2 mmHg to 11.1 ± 4.3 mmHg, *p* < 0.01). A generalized linear model demonstrated that the final IOP, mean deviation, and pattern standard deviation of the VF were positively affected by the systemic PEA treatment (*p* < 0.01). The conclusion was that PEA reduces IOP and improves visual field indices in individuals affected by NTG. During the study, no side effects were documented [9]. Lastly, in a small controlled pilot trial in 15 patients, PEA lowered the laser iridectomy induced raised IOP compared to placebo, and patients were pretreated with PEA (600 mg/day) or placebo for 2 weeks [12].

In all these studies the effects supported PEA's ocular pressure reducing effects and/or were suggestive for its retinoprotective effects. Although the dose in the studies described above was 600 mg PEA/day, we recommend the double dose (1200 mg/day) based on practice and due to the fact that various clinical trials dosed at a higher dose ranges, and for PEA there are no dose limiting side effects up to 2400 mg/day. Even doses as high as 2400 mg/day have been used in our clinic in hundreds of patients and are free of such side effects. Furthermore, there are strong pharmacological and clinical indicators for a linear dose effect curve, both in clinical and in preclinical studies, 300–600 mg PEA/day being the low effective dose. Therefore, a higher dose most probably has a greater chance of being effective. A full dose-response study in glaucoma hopefully will be organized in the future.

The mechanism behind PEA's effect on the ocular pressure has been explored using a porcine anterior segment-perfused organ culture model [47]. In that model, PEA caused a concentration-dependent enhancement of outflow facility, with the maximum effect achieved at a low concentration of 30 nM of PEA [47]. PEA also has a number of cell protective properties, and those combined mechanisms could have a significant relevance in the long term treatment of glaucoma. Some of the following findings are supportive for this idea.

Recently, new insights have emerged related to the function of lipid signaling compounds such as PEA in the migration of retinal projections towards their targets [48]. In addition to this a hydrolysis inhibitor, the FAAH inhibitor URB597, of PEA could induce retinal ganglion cell neuroprotection after optic nerve axotomy [49]. Furthermore, PEA and its related molecules could alleviate overactive innate immune responses in Müller glia, and the explorers of that paradigm stated that such therapy may therefore orchestrate a molecular switch to influence the balance of pro- and anti-inflammatory cytokine generation in such a way to create, as they called it, “a prosurvival milieu” [50]. The clinical results described above are supported by a vast number of preclinical reports, documenting the anti-inflammatory, neuroprotective properties of PEA and more specifically its retinoprotective properties. In the next two paragraphs some of this evidence will be discussed. The important research question about whether glaucoma affects the metabolism of PEA in the retina is still unanswered. Our hypothesis would be that due to the chronic inflammation either the synthesis of PEA is decreased or its metabolism is increased.

## 3. Palmitoylethanolamide: Early Insights in Anti-Inflammatory and Neuroprotective Properties

**Figure 1:**
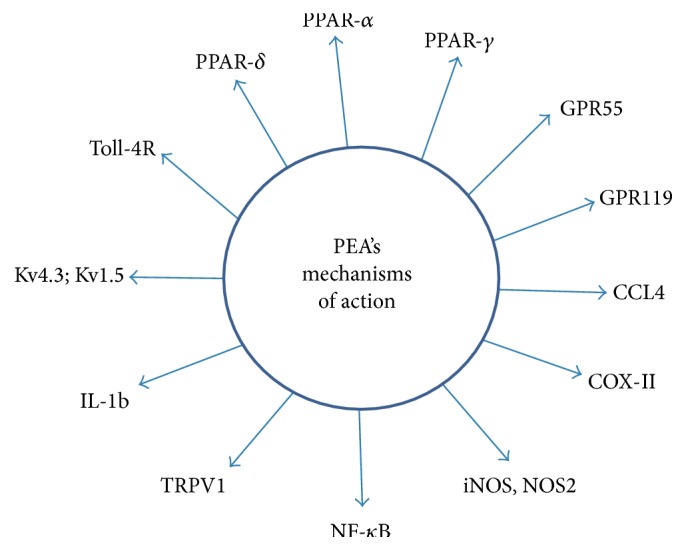
Different molecular targets of PEA. PPAR: peroxisome proliferator activated receptor; GPR-55: 119-orphan G-protein coupled receptors; CCL: chemokine ligand; COX: cyclooxygenase; iNOS: inducible nitric oxide synthase; TRPV: transient receptor potential cation channel subfamily V; IL: interleukin; Kv1.5,4.3: potassium voltage gated channels; Toll-4 R: toll-like receptor.

PEA is an endogenous evolutionary old molecule that probably came into existence more than 300 million years ago. It can be found in sea urchins and shellfish as well as humans [51]. PEA is a pleiotropic mediator and modulator, having an effect on several receptors (Figure 1) [52]. Its efficacy and tolerability have been documented in over 40 clinical trials in around 5000 patients and its side-effect profile is very benign [53, 54]. However, due to the fact that PEA is available as a nutraceutical, its therapeutic potential is not well recognized in the medical field yet. PEA however has been proven to be useful in the clinical treatment of chemotherapy induced neuropathy and in a number of nerve compression syndromes supporting PEA's neuroprotective and restorative properties [53–55].

Palmitoylethanolamide is an endogenous signaling lipid in possession of clear anti-inflammatory properties [56]. Its value as a prophylactic and therapeutic agent for flu and common cold was evaluated in the 70s of the 20th century [57–59]. During this period, it also became clear that PEA was synthesized in many different tissues. Decades ago, it is understood that PEA and related N-acetylethanolamines protect cells against insults, such as ischemia, immunological pathologies, and damage. For instance, PEA accumulates in infarcted tissue, while it could not be detected in the healthy heart and brain tissues [60]. PEA and related lipids were also documented to exist in different vertebrates, even in invertebrates, as well as in a great number of organs, including the brain and the spinal cord [61, 62]. In 1993, the Nobel laureate Rita Levi-Montalcini shed new light on how PEA could achieve its specific properties in her milestone paper [63]. In that paper, she described a number of experiments where orally administered PEA to rats could inhibit overactive mast cells (MCs) and reduce local inflammatory markers such as tumor necrosis factor-*α* (TNF-*α*). Few years later, she was the first to discuss PEA's organ specific, neuroprotective properties, and PEA's mechanism of action [64]. Levi-Montalcini and coworkers were also first to propose the ALIA mechanism or the “Autacoid Local Inflammation Antagonism,” later modified into “Autacoid Local Injury Antagonism” and defined PEA as such an “ALIAmide.” An ALIAmide is an autacoid synthesized and released in response to injury or inflammation acting locally to counteract the pathological events [65, 66]. However, in some chronic inflammatory states, PEA tissue levels can also be decreased, either due to decreased synthesis or due to enhanced metabolism [66]. Compounds such as PEA thus can be seen as endogenous protective and reparative molecules. Her work however did not lead to the identification of the main molecular target of PEA. Only many years later the link between these properties and the effect of PEA on the nuclear receptor PPAR-*α* was understood, based on the seminal work of Piomelli and LoVerme and colleagues [67, 68].

## 4. Palmitoylethanolamide: Retina-Protective Properties

As the retina consists of nervous tissue, neuroprotective properties of molecules can also be regarded as an indication for retinoprotective activity. N-Acetylethanolamines (NAEs) are lipophilic, autocrine signaling molecules formed in all tissues on demand from membrane phospholipids called N-acylated-phosphatidylethanolamines. The notable examples include PEA, oleoylethanolamide, stearoylethanolamide, and anandamide (arachidonoyl-ethanolamide (AEA)). PEA, a structural relative of anandamide, is a saturated NAE (C16:0) with a palmitoyl moiety. PEA and AEA are also present in peripheral tissues as well as in the central nervous system, with the former being ten times more abundant [69]. Specific noxious stimuli may lead to their independent accumulation. For instance, in an animal model of autoimmune encephalomyelitis, there was a clear increase in PEA levels [70].

Many of the known biological properties of PEA pointing to its putative retina-protective properties can be explained through the dose dependent modulation of peroxisome proliferator-activated receptors, especially the PPAR-*α* receptor. The numbers of PEA targets are currently increasing. Recently it was described that PEA inhibits the induction of the proinflammatory genes IL-1b, CCL4, and NOS2 and adhesion molecules such as ICAM-1 and P-selectin [71]. CCL4 can induce monocyte migration and this migration is also blocked by PEA [72]. The receptor system however that mediates the majority of the agent's effects in the case of PEA is due to activation of PPAR-*α* and downstream secondary targets such as NF-*κ*B, TNF-alpha, Il1b, and ICAM, as listed in Figure 1. Evidence for roles of GPR55 and GPR119 however remains scant. Figure 1 shows a number of known targets of PEA.

Both PPAR-*α* and PEA are present in nervous tissue and their expression may show large changes during pathological conditions. Currently there seems to be a consensus in the literature regarding the idea that the PPAR family is one of the primary targets of PEA. Through activation of the PPARs, especially PPAR-*α*, PEA is attenuating proinflammatory mediators and/or increasing anti-inflammatory mediators [42, 73]. However, the role of PEA is not thought to be limited to the action on PPAR-*α*, as different targets such as PPAR-*δ* and PPAR-*γ* clearly play an additional role. In fact, a study conducted on spinal cord injured mice showed that PEA treatment induced a limited infiltration of inflammatory cells, a protective effect that was attenuated after treatment with PPAR-*γ* and PPAR-*δ* antagonists. PEA increases PPAR-*α* and PPAR-*γ* expression, thus restoring any neuroinflammation induced downregulation of these receptors [39]. Activation of PPAR-*δ* inhibited streptozotocin-induced diabetic nephropathy through anti-inflammatory mechanisms [74]. PEA was also seen to show anti-inflammatory effects in nonneuronal tissues. For instance, it downmodulates skin MC activation and hence reduces immunologically induced release of histamine, PGD_2_, and TNF*α* [75]. PEA reduces spinal cord inflammation and tissue injury with significant reduction of nitrotyrosine formation, proinflammatory cytokine expression, NF-*κ*B activation, inducible nitric-oxide synthase expression, and apoptosis [46]. Colon inflammation was also seen to be improved by PEA, and proinflammatory markers and macroscopic signs of ulcerative colitis were decreased [33]. Downstream inhibition of NF-*κ*B by PPAR-*α* was responsible for the effects, as the effects were abolished by a specific antagonist. PEA is also able to upregulate the various PPAR receptors in damaged tissue [76]. These upregulated and activated receptors are key for many downstream effects of PEA. PEA at a dose of 10 mg/kg intraperitoneally protected nigrostriatal neurons from neurotoxicity and neuroinflammation [43]. PEA remains neuroprotective even when administered once the insult has been initiated. PEA had shown superior neuroprotective activity compared to minocycline (TNF-*α* agonist) in models of neurotoxicity [43]. In mice models of Alzheimer's disease, PEA reduced the increased lipid peroxidation, protein nitration, inducible nitric oxide synthase expression, and the induction of proapoptotic pathways induced by amyloid injection [77]. PEA counteracted the negative effects of amyloid on cell viability in cultured cortical neurons and astrocytes [26]. Despite the fact that PPAR-*α* is the most probable target for these effects, PPAR-*γ* and PPAR-*δ* were shown also to contribute to the neuroprotective role. PPAR-*γ* and PPAR-*δ* presence is reported in spinal cord under physiological conditions. In one study, 24 hours after spinal cord injury, PPAR-*γ* and PPAR-*δ* expression was significantly reduced in spinal cord homogenates. PEA treatment could significantly restore these decreases again to normal basal levels. Therefore, the anti-inflammatory and protective effects of PEA are favored by the presence of PPAR-*α*, PPAR-*δ*, and PPAR-*γ* receptors at various sites [39].

The putative retinoprotective properties of PEA are based on the same mechanisms as the neuroprotective and cytoprotective properties. In Table 1 an overview is given of studies supporting PEA's cytoprotective properties.

## 5. PEA and Its Putative Relevance for the Treatment of Diabetic Retinopathy

Diabetic retinopathy is the most common microvascular complication of diabetes mellitus and the main cause of blindness within the working-age population in industrialized nations [78]. More recently new pathways which may be involved in the pathogenesis of diabetic retinopathy have been identified, such as inflammation, nerve growth factor autophagy, and epigenetics. Epidemiologic studies have shown an association between the appearance of inflammatory biomarkers and the occurrence of type 2 diabetes mellitus and its complications [79]. Diabetics have increased levels of inflammatory markers, including C-reactive protein (CRP), interleukin-6 (IL-6), and TNF-*α* [80]. There is increasing evidence that inflammatory processes play a considerable role in the pathogenesis of DR [81].

PEA most probably produces retinoprotection via its anti-inflammatory properties we described above, preventing oxidant stress and inhibition overactivation of Müller cells mircoglia and asterocytes. This activation of the innate immune response in the retina is thought to be triggered by advanced glycation end products (AGE) [82]. The retinal Müller cells are the major source of inflammatory cytokines in the retina [83]. But microglia and asterocytes also play a role [84]. The hyperglycemia leads in many cases to an increased production of proinflammatory cytokines by the Müller cells and asterocytes, such as IL-1*β* [85]. PEA has been found in the past to inhibit overactivation of various glia cells. In a rat astrocyte model, PEA downregulates reactive gliosis by reducing proinflammatory molecules and cytokine release through the inhibition of NF-*κ*B. PEA causes a marked reduction of astrocyte activation and provides neuronal protection in both mixed neuroglial and organotypic hippocampal cultures [27]. These findings are relevant, as retina glia cell activation is also thought to be an important pathogenetic element not only in diabetic retinopathy but also in glaucoma [28, 86].

Toll-like receptors (TLRs) play also an important role in innate immune responses and inflammation and the pathogenesis of diabetic retinopathy [87]. TLR-4 for instance is upregulated in hyperglycemia and TLR-4 activation and this activation leads to increased inflammation possibly via reactive oxygen species (ROS) and the authors suggested this pathway also could contribute to DR. In different paradigms, the same pathogenetic role for TLR-4 in DR has been postulated [88]. TLR-4 polymorphisms are also suggested to increase the risk for DR in Diabetes type 2 patients [89]. PEA inhibits TLR-4 activity and downregulates TLR-4 triggered inflammation cascades [33].

PEA thus has the potential to inhibit these various proinflammatory activated cascades, which seems relevant to the treatment of both diabetic retinopathy and glaucoma.

## 6. Conclusion

PEA's safety and efficacy have been evaluated in a number of clinical trials in inflammatory conditions and chronic pain states, including glaucoma. PEA has been shown to be safe and effective up to 1.8 gm/day [58], due to its mechanisms of action and remarkable safety, without any documented drug-drug interaction. Due to the convergent pathogenetic pathways related to retinal glia activation, PEA holds a promise for both glaucoma as well as diabetic retinopathy.

PEA is a pleiotropic naturally occurring endogenous N-acetylethanolamine that plays an important biological role in many living organisms, including humans. Its beneficial effects are dose dependent and mediated through various receptors such as PPAR-*α*, PPAR-*γ*, PPAR-*δ*, GPR 119, and TRPV1. PEA is known to downregulate proinflammatory genes and possess a broad anti-inflammatory, antioxidant, and cytoprotective activities. As both glaucoma and diabetic retinopathy share a common pathogenetic pathway, PEA should be considered in the treatment and prophylaxis of retinal damage in both disorders.

## Figures and Tables

**Table 1:** Summary of preclinical studies related to PEA's cytoprotective effects.

Year	Dose PEA	Main results	Reference
2015	5 *μ*M in vitro	Inhibition of the Ca^2+^-dependent release of glutamate	[24]
2015	5 mg/kg	Diminished inflammation, demyelination, axonal damage, and inflammatory cytokine expression in a multiple sclerosis model	[25]
2015	0.1 *μ*M in vitro	Protection cell viability in cultured cortical neurons and astrocytes against inflammation	[26]
2015	10^−8^–10^−6^ M	Concentration-dependently reduced expression of proinflammatory and proangiogenic markers	[27]
2014	1 mg/kg	Prevention of induced afferent mechanical sensitization	[28]
2014	200, 400 and 800 *µ*g/mL	Inhibition of inflammation markers and chymase expression in granulomatous tissue	[29]
2014	30 mg/kg sc	Increased AMP-activated protein kinase-*α* phosphorylation and carnitine palmitoyltransferase 1 transcription in adipose tissue; polarized adipose tissue macrophages to M2 lean phenotype	[30]
2014	10 mg/kg	Reduction of structural radiation injury, intestinal wall thickness, collagen deposition, intestinal inflammation, and increased anti-inflammatory IL-10 and IL-6	[31]
2013	10 mg/kg	Reduction of the clinical signs of type II collagen-induced arthritis as well as of paw edema compared to control	[32]
2013	2, 10 or 50 mg/kg	Improves all macroscopic signs of colitis and decreases the expression and release of all the proinflammatory markers	[33]
2013	30 mg/kg	Reduction of hypertension and protects kidney injury	[34]
2013	1 *μ*M control in vivo	Protected SCI-associated neuroinflammation in vivo and in vitro	[35]
2013	0.1 *µ*M, in vitro	Rescue of neuron damage by amyloid and reduction of neuroinflammation (decrease of astrocyte activation)	[36]
2013	5–10 mg/kg	Normalizing the activity of sensitized nociceptive neurons; significant reduction of mechanical allodynia and thermal hyperalgesia in a dose-dependent manner	[37]
2013	10 mg/kg	Strong reduction of microglia activation and PEA delayed mast cell recruitment, protection of mast cells against degranulation, and abolition of the nerve growth factor increase, reducing pain	[38]
2013	10 mg/kg	Protection of spinal cord damage; restoration of PPAR-*δ* and PPAR-*γ* expression in spinal cord after damage	[39]
2013	10, 20, 40, 60 mg/kg i.p.	Showing anti-epileptic properties in a rat model	[40]
2013	NR	Blunted A*β*1-42-induced neurotoxicity and controlled glial activation	[41]
2012	10 mg/kg	Significant attenuation of the degree of renal dysfunction, injury, and inflammation caused by ischemia-reperfusion injury	[42]
2012	10 mg/kg	Reduction of MPTP-induced microglial activation, the number of GFAP-positive astrocytes, and reduction of neutrophil infiltrations, reduction of TNF-*α*, IL-1*β* and iNOS in spinal cord and prevention of SCI-induced I*κ*B-*α* degradation and Bax expression	[43]
2012	10 mg/kg	Reduction of apoptosis, brain infarctions, and various inflammatory parameters	[44]
2011	10 mg/kg	Significant reduction of mast cell infiltration, expression of mediators like NGF, the activation of microglia, and astrocytes expressing cannabinoid CB(2) receptor after spinal cord injury	[45]
2008	10 mg/kg	Significant reduction of spinal cord inflammation and tissue injury, neutrophil infiltration, and proinflammatory cytokine expression and significant amelioration of the recovery of motor limb function	[46]

NR: Nonreported.

## List of Abbreviations

AEA: Arachidonoyl-ethanolamide
DR: Diabetic retinopathy
IOP: Intraocular pressure
MC: Mast cell
NAE: N-Acetylethanolamine
PEA: Palmitoylethanolamide
PPAR: Peroxisome proliferator activated receptor
ROS: Reactive oxygen species
TNF-*α*: Tumor necrosis factor-*α*.
